# Association of specific HLA alleles and haplotypes with pemphigus vulgaris in the Bulgarian population

**DOI:** 10.3389/fimmu.2022.901386

**Published:** 2022-08-02

**Authors:** Kossara Drenovska, Milena Ivanova, Snejina Vassileva, Martin Abu Shahid, Elissaveta Naumova

**Affiliations:** ^1^ Department of Dermatology and Venereology, University Hospital “Alexandrovska”, Medical Faculty, Medical University - Sofia, Sofia, Bulgaria; ^2^ Department of Clinical Immunology, University Hospital “Alexandrovska”, Medical Faculty, Medical University – Sofia, Sofia, Bulgaria

**Keywords:** pemphigus vulgaris, Bulgarian patients, HLA haplotypes, predisposing role, decreased allele frequency

## Abstract

Pemphigus vulgaris (PV) is an autoimmune bullous dermatosis with uneven geographic distribution and higher incidence in certain populations. In previous studies, a relatively high incidence of PV was reported in Bulgaria (0.47/100,000/year) comparable to that in other countries. The genetic background was considered responsible for the disease susceptibility, and multiple reports have proven PV to be an HLA-associated condition. The aim of our study was to analyze the role of genetic factors in the development of PV in Bulgaria. HLA genotyping was performed in 56 PV patients, ethnic Bulgarians whose diagnosis was confirmed based on clinical, histological, and immunofluorescent findings. The control group consisted of 204 healthy individuals from the Bulgarian population without evidence for HLA-associated autoimmune diseases. HLA-A,-B,-DRB1,-DQB1 analysis was performed by PCR-SSP. Our results revealed predisposing associations with DRB1*14, DRB1*04:02, and B*38, B*55, while allele DRB1*03:01 and the corresponding haplotypes were significantly decreased in the PV patients. The predisposing role of these alleles has been observed in other populations. All reported predisposing DRB1 alleles have the same amino acids at key positions of the beta chain of the HLA molecules, 26 (Phe), 67 (Leu or Ileu), 70 and 71 (hydrophobic AA: Gln, Arg, Asp, or Glu), and 86 (Val), which is important for the selective presentation of desmoglein 3 peptides. Additionally, specific alleles HLA-A*01 and DRB1*11 were identified with decreased frequencies in the patients’ group, the last one being a common protective allele for autoimmune diseases in the Bulgarian population. The elucidation of the role of genetic factors for the development of pemphigus will help explain its higher incidence and clinical variability in certain populations.

## Introduction

Pemphigus encompasses a group of organ-specific autoimmune blistering diseases of stratified squamous epithelia characterized by the formation of blisters and erosions on the mucous membranes and/or the skin (1). The hallmark of pemphigus are the immunoglobulin G (IgG) autoantibodies raised against desmosomal cadherins, of which desmogleins (Dsg)1 and Dsg3 are best characterized and considered as most important (2). The binding of autoantibodies to their target antigens on the keratinocyte surface leads to loss of intercellular adhesion resulting in acantholysis and formation of intraepithelial blisters.

Based on clinical, histologic, and immunopathologic findings, pemphigus is classically divided into two major subtypes: pemphigus vulgaris (PV) and pemphigus foliaceus (PF) (3, 4). In PV, acantholysis occurs deep in the epidermis just above the basal-cell layer and is associated with autoantibodies to Dsg3, which is mainly expressed in the mucous membranes and lower epidermal layers of the skin. In PF, the blisters are high in the epidermis below the stratum corneum and are associated with autoantibodies to Dsg1, which is expressed throughout the epidermis in the skin, and to a much lesser extend in the mucous membranes (5, 6). The profile of autoantibodies corresponds to specific clinical features, i.e. presence of painful muco-cutaneous blistering in PV *via* production of antibodies to Dsg3 and Dsg1, and involvement of the skin only *via* production of antibodies to Dsg1 in PF, respectively (7).

Incidences of the various forms of pemphigus vary in the different ethnicities and from country to country (5). PV is most common in Europe and the USA where its frequency is showing rates of 65 – 90%, whereas PF is the prevalent subtype in Northern Africa and Southern America (8). In addition, PF exists as sporadic cases distributed worldwide, but also in the form of endemic foci in well-defined regions of South America (Brazil and Northern Columbia) and North Africa (Tunisia, Morocco) (8–11).

PV is the more severe pemphigus variant affecting mostly middle-aged patients with peak in the 4th-6th decades, while children and elderly are exceptionally involved. PV is known for its uneven geographic distribution and higher incidence in certain populations, namely Ashkenazi Jewish and people from Mediterranean origin (12). Respectively, the highest annual incidence rates of PV are reported in Tehran District, Iran (16.0 / million) (13), among the Jewish population in Jerusalem, Israel (16.1 / million) (14), and Connecticut (32.0 / million) (15). For comparison, in Western countries sporadic PF has shown and estimated annual incidence of < 1 case per million (8, 11). In Europe, various rates have been reported, ranging between 0.8/million/year in Finland (16), 1.7/million/year in France (10), and 6.0 in Sicily (17).

Mediterranian coutries seem to occupy a particular place in the epidemiology of PV (14, 18, 19). The same implies to a large extent for the South-Eastern European countries, more precisely located on the Balkan Peninsula, where PV shows an overall high incidence rates (**Table 1**) (18–27). The Balkan countries are known for their multi-ethnic populations comprising Slavic caucasians, Albanians, Gipsies, ethnic Turks, Armenians, etc. It is of note that the highest PV rates have been found among Roma people (Gipsies) reaching rates of 24/million/year (22). In addition, an epidemiologic study from Germany found a nine-fold higher incidence of PV among non-German foreigners (from Turkey and Italy), i.e. 6.8/million/year, compared to native Germans (0.8/million/year) (28).

**Table 1:** Incidence of pemphigus vulgaris in Southern-European (Balkan) countries.

Country	Annual incidence rate/million inhabitants	Reference
Jerusalem, Israel*	16.1	Pisanti et al., 1974 (14)
Sofia, Bulgaria	4.7	Tsankov et al., 2000 (18)
South Vojvodina, Serbia Central Serbia	6.6 4.0	Goluŝin et al., 2005 (20) Milinković et al., 2016 (21)
Skopje, Northern Macedonia	4.4	V’lckova-Laskoska et al., 2007 (22)
Zagreb, Croatia	3.7	Marinovic et al., 2011 (23)
Northwestern Romania	4.0	Baican et al., 2010 (24)
Wurzburg and Mannheim, Germany	0.8 native Germans 6.8 foreigners**	Hahn-Ristic et al., 2002 (28)
Thessaloniki, Greece Greece	8.0 9.3	Michailidou et al., 2007 (25) Kyriakis et al. 1989, 1995 (19, 26)
Turkey***	4.7	Yaili et al. 2017 (27)

*Included for comparison purposes.

**Southern Europeans living in Germany.

***No data are available from the European part of Turkey, but the latter is included because >1 million of ethnic Turks continue to inhabit the Balkan countries since they first settled there during the Ottoman period.

The etiology of pemphigus is largely unknown. Among the factors discussed are environmental agents, infections, drugs, and tumors. Genetic background was also considered responsible for the disease susceptibility and multiple reports have proven PV to be a HLA-associated condition. PV has been associated both with classical HLA class I and class II alleles (29, 30), and non classical genes such as HLA-G and the transporter associated processing gene (29, 30). Strong association has been found with certain HLA alleles and haplotypes throughout the world, namely HLA class II HLA-DRB1*04:02, DQB1*03:02 have been found in Jewish patients and HLA-DRB1*14:01/14:04, DQB1*05:03 in non-Jewish patients (32).

Therefore, the well-known increased prevalence of PV in distinct ethnic groups such as Jews, Iraqi, Indians, and Iranians could be explained with the high frequency of specific predisposing for PV HLA alleles.

Only scarce data are available on HLA in PV in Eastern and South-Easthern European countries and namely those located at the Balkan Peninsula. The only data come from Slovakia (30, 33), Serbia (34), and Turkey (29). Previous studies among Bulgarian patents with pemphigus carried out using serologic HLA-typing have found increased frequencies of HLA-A9, A10, B13, DR4, DR5, DQ2, and DQ3 specificities, as well as low frequency of HLA-A3, B8, DR1, and DR3, pointing out the protective role of the latter on the development of the disease (35). However, both patients with PV and PF were included in this earlier series. Given the fact that PV is the prevalent pemphigus variant in Bulgaria, we decided that it would be worth to focus our study of the genetic markers of pemphigus by selecting patients with PV only.

## Objective

The aim of the present study was to analyze the role of HLA alleles and haplotypes for the development of PV among the Bulgarian population.

## Material and methods

### Patients

The study was conducted prospectively and included pemphigus patients from Bulgaria, hospitalized in the Department of Dermatology and Venereology, University Hospital “Alexandrovska”, Medical University – Sofia, during the period January 2009 – December 2011 (**Table 2**). Fifty-six patients with PV were enrolled, 19 men (34%), and 37 women (66%), with age range 15-80 years (mean age 49.98 ± 15.4). All patients were ethnic Bulgarians, with both parents and grandparents born in the country. The diagnosis of PV was confirmed based on clinical, histopathologic, and immunofluorescence criteria, i.e. presence of bullae and/or erosions on the skin and/or mucous membranes, suprabasal acantholytic blisters upon histology, and positive direct immunofluorescence microscopy on perilesional skin showing deposition of IgG on the epithelial cell surface producing the typical epidermal intercellular staining pattern; besides, most patients sera tested positive for pemphigus antibodies by indirect immunofluorescence on monkey esophagus substrate.

**Table 2:** Demographic and clinical data. PV patients and healthy controls from the Bulgarian population.

	PV patients	Healthy controls
**Number studied**	56	204
Sex distribution*		
Males	19 (34%)	100 (49%)
Females	37 (66%)	104 (51%)
M : F	1:1.95	1:1.04
Age distribution*		
Range	15 - 80	20–46
Mean	49.98 ± 15.4	31.5 ± 10.6
Median	47.5	33

*P-values (t-sided and chi-square test) > 0.005—non-significant.

### Controls

The control group consisted of 204 unrelated healthy individuals from the Bulgarian population: 100 men (49%) and 104 women (51%) with age range 20-46 years (mean age 31.5 ± 10.6) without family history of autoimmune diseases.

### Ethics

All patients and healthy controls provided informed consent for testing performed as part of the institutional review boards approved standard operating procedures at our institutions. The principles of the Declaration of Helsinki were strictly followed during the study.

### HLA typing

HLA-tests for all patients and controls were performed at the Department of Clinical Immunology, University Hospital “Alexandrovska”, Medical University – Sofia.

Genomic DNA was extracted from peripheral blood on an iPrep Purification Instrument (Thermo Fisher Scientific, USA) with iPrep PureLink gDNA Blood Kit (Thermo Fisher Scientific, USA). HLA-A,-B,-DRB1,-DQB1 genotyping was performed by high resolution PCR-SSP method using Olerup-SSP typing kits (Olerup^TM^, Quiagen, Germany) according to the manufacturer’s instructions. High-resolution PCR-SSP typing was performed for allele groups HLA-DRB1*03 and DRB1*04 by Olerup-SSP typing kits.

### Statistical analysis

HLA allele and haplotype frequencies were estimated using Expectation-Maximization (EM) algorithms *via* Arlequin program (version 1.1). Comparative analysis between the groups was performed using Chi-square method. Bonferroni correction for multiple tests was applied by multiplying “*p”* by the number of comparisons. Probability values were considered significant if *pc* < 0.05. Odds ratio (OR) estimate was calculated using the methods of Woolf and Haldane with modification for small numbers (36). Ninety-five percent confidence intervals were given.

## Results

Concerning the demographic distribution of patients and controls no statistically significant differences were detected (**Table 2**). Comparisons of HLA allele frequencies between patients and controls showed statistically significant association with one HLA-A, two HLA-B, and four HLA-DRB1 alleles (**Table 3**).

**Table 3:** HLA alleles significantly associated with PV in the Bulgarian population.

HLA allele	Allele frequency		OR	HLA allele	Allele frequency		OR
	Patients	Controls			Patients	Controls	
DRB1				HLA-B			
03:01*	0.0234	0.0931	0.23	38***	0.1250	0.0294	4.33
04:02***	0.2730	0.0231	14.8	55***	0.0859	0.0147	6.30
11***	0.0075	0.2034	0.03	HLA-A			
14***	0.2187	0.0613	3.38	01*	0.0234	0.0900	0.21

*Рс <0.05 ***Рс <0.001.

Our results revealed predisposing associations with DRB1*14, DRB1*04:02, B*38, and B*55 (**Table 3**). Additionally, HLA-A*01, HLA-DRB1*03:01 and DRB1*11 showed negative association with PV in our population. This observation is interesting since the DRB1*11 allele has been previously described as being protective for autoimmune diseases in the Bulgarian population (37, 38).

Comparisons of amino acids (AA) sequences of the portions encoded by predisposing and protective alleles observed showed that all predisposing alleles have the same amino acids (АА) at key positions of the beta chain of the HLA molecules: 26 (Phe), 67 (Leu or Ileu), 70 and 71 (hydrophobic AA: Gln, Arg, Asp or Glu), and 86 (Val). This might be important for the selective presentation of Dsg3-peptides (**Table 4**).

**Table 4:** Amino acid positions in HLA-DRB1 alleles associated with pemphigus vulgaris in the Bulgarian population.

HLA allele	AA positions				
	26	67	70	71	86
**Predisposing**					
DRB1*04:02	F	I	D	E	V
DRB1*14	F	L	R	R	V
					
Protective					
DRB1*11	F	F	D	R	V
DRB1*03:01	Y	L	Q	K	V

**АА**, amino acid.

Additionally, we compared the distribution of HLA-DRB1-DQB1 and HLA-A-B-DRB1 haplotypes between patients with PV and healthy controls (**Tables 5**, **6**). In agreement with HLA-DRB1 allele distribution, two haplotypes, HLA-DRB1*04:02/DQB1*03 and DRB1*14/DQB1*05 were positively associated with PV and therefore, considered as predisposing, while the haplotypes DRB1*03:01 / DQB1*02 and DRB1*11/DQB1*03 showed significantly decreased frequency in the patients’ group (**Table 5**).

**Table 5:** HLA-DRB1-DQB1 haplotypes associated with pemphigus vulgaris in the Bulgarian population.

Haplotype	Haplotype frequency		OR
	Patients n=56	Control n=204	
DRB1*03:01 DQB1*02 *	0.0234	0.0904	0.24
DRB1*04:02 DQB1*03 ***	0.2730	0.0231	14.86
DRB1*11 DQB1*03 *	0.0937	0.2409	0.10
DRB1*14 DQB1*05 ***	0.2187	0.0422	6.60

*Рс <0.05 *** pс <0.001.

**Table 6:** HLA-A-B-DRB1 haplotypes associated with pemphigus vulgaris in the Bulgarian population.

Haplotype	Haplotype frequency		OR
	Patients n=56	Controls n=204	
A*02 B*49 DRB1*14 *	0.02778	0.00000	11.49
A *02 B*15 DRB1*04:02 *	0.02778	0.00000	11.49
A *03 B*38 DRB1*04:02*	0.02778	0.00000	11.49
A *03 B*51 DRB1*04:02 *	0.02778	0.00000	11.49
A *24 B*44 DRB1*04:02 **	0.06944	0.00735	10.07
A *25 B*18 DRB1*04:02 *	0.02778	0.00000	11.49
A *31 B*51 DRB1*04:02 *	0.02778	0.00000	11.49

*Рс <0.05 **Pc <0.01.

Analysis of three–loci haplotypes showed that seven haplotypes, i.e. one DRB1*14 haplotype and six DRB1*04:02 haplotypes, were observed with statistically significant increased frequency in patients compared to the controls (**Table 6**).

## Discussion

In the present study we analyzed for the first time HLA allele and haplotype association in Bulgarian patients with PV using population based approach. Analyzing DRB1 alleles we observed a strong association with DRB1*04:02 and less marked relation to DRB1*14. These data are in accordance with previously published reports worldwide, including studies among various South-European populations, such as in Italy (39), Sardinia (40), Spain (40), France (42), in Ashkenazi Jews (43, 44), as well as in Asia (45) and South America (46–48). In the Bulgarian population, DRB1*03:01 allele demonstrated negative association with PV, similarly to Sardinians (40). Analogous results for DRB1*11 support similar findings in various autoimmune diseases in the Bulgarian population (37, 38). In contrast to other authors (40, 49–51) we could not confirm association related to DQB1 genes in our study. Moreover, two alleles of the HLA-B locus, namely B*38 and B*55, were related to a statistically significantly increased risk for the development of PV among Bulgarians. The predisposing association related to the presence of B*38 is in accordance with the results from studies among Jews (49–51), Sardinians (40), and Iranians (52). On the other hand, B*55 was reported to be predisposing in white non-Jewish Europeans and Iranians. It is interesting to note the statistically significant negative association for HLA-A*01 allele observed in the present study.

Identically to other populations and corresponding to the known linkage disequilibrium, in patients from Bulgaria, two haplotypes were found to be related with higher risk for the development of PV, namely DRB1*04:02-DQB1*03, and DRB1*14-DQB1*05. On the contrary, DRB1*03:01-DQB1*02 haplotype demonstrated negative association, similarly to the results among Sardinian population (40). What was more specific for our population was the additional finding on the decreased frequency of DRB1*11-DQB1*03 in PV in contrast to other authors who detected participation of DRB1*11 and DQB1*03:01 alleles in the presentation of Dsg3 and in the disease predisposition, respectively (40). The absence of a separate association between DQB1 genes and PV in the Bulgarian population confirms the hypothesis for the dominating role of DR genes. This is in agreement with the hypothesis on the role of DR molecules in the presentation of Dsg3 peptides to autoreactive T-cells (54).

Seven haplotypes (6 DRB1*0402; 1 DRB1*14) were detected with statistically significant frequency in PV patients compared to controls: A*02-B*15-DRB1*04:02; A*03-B*38-DRB1*04:02; A*03-B*51-DRB1*04:02; A*24-B*44-DRB1*04:02; A*25-B*18-DRB1*04:02; A*31-B*51-DRB1*04:02; A*02-B*49-DRB1*14. The predisposing role of A*03-B*38-DRB1*04:02 haplotype in Bulgarian population is in accordance with the haplotype associations reported among Ashkenazi Jews with pemphigus.

It is hypothesized that the role of HLA allele variants in the pathogenesis of PV is related to the desmoglein peptides of specific autoreactive CD4+ T-cell clones (55). A common immunodominant epitope initiates a T-cell response that may change through epitope spreading. All alleles observed in our study as predisposing to PV demonstrate specific AA portions at key positions: 26 (Phe), 67 (Leu or Ileu), 70, 71 (hydrophobic amino acids: Gln, Arg, As, or Glu), and 86 (Val) in the DR beta-chain. It was proved that the negative load of P4- domain (pocket) of DRB1*04, formed by glutamate at 71 beta-position, is necessary for the selective presentation of Dsg3 peptides. Therefore, this is in agreement with the hypothesis on the role of DRB1 alleles in autoantigen presentation and pathogenesis of PV.

Immune tolerance to epidermal antigens, participating in the development of pemphigus, may be called “immune ignorance” more than tolerability of the central and peripheral immune system due to the fact that autoreactive T-cells are present in healthy individuals as well. The autoimmune reaction may be triggered through molecular mimicry between xenoantigen (infectious agent or drug), modified autoantigen (drug) or tumour antigen, and autoantigen-target (56).

Studying the target antigens and their corresponding autoantibodies would help the diagnostic of pemphigus subtypes and the prognostic evaluation for the patient.

The elucidation of the role of genetic factors for the development of pemphigus will help explain its higher incidence and clinical variability in certain populations.

Getting familiar with genetic predisposition and the triggering environmental factors, together with developing recombinant antigens for detection of autoreactive B-cells, may help the development of specific immunotherapy of pemphigus (57).

## Conclusions

HLA allele and haplotype distribution among Bulgarian patients with PV is similar to the already established predisposing associations with DRB1*14, DRB1*04:02, B*38, B*55, whereas DRB1*03:01 alleles and the corresponding haplotypes were significantly decreased in PV patients from the Bulgarian population. We also report specific negative HLA associations for the Bulgarian population, namely DRB1*11 and A*01 but additional studies are necessary to further demonstrate a direct preventive benefit of carrying these alleles.

## Data availability statement

The original contributions presented in the study are included in the article/supplementary material. Further inquiries can be directed to the corresponding author.

## Ethics statement

Ethical review and approval was not required for the study on human participants in accordance with the local legislation and institutional requirements. The patients/participants provided their written informed consent to participate in this study.

## Author contributions

All authors listed have made a substantial, direct, and intellectual contribution to the work, and approved it for publication.

## Conflict of interest

The authors declare that the research was conducted in the absence of any commercial or financial relationships that could be construed as a potential conflict of interest.

## Publisher’s note

All claims expressed in this article are solely those of the authors and do not necessarily represent those of their affiliated organizations, or those of the publisher, the editors and the reviewers. Any product that may be evaluated in this article, or claim that may be made by its manufacturer, is not guaranteed or endorsed by the publisher.
